# Anterograde and trans-synaptic neurodegeneration in aquaporin-4-antibody neuromyelitis optica spectrum disorder patients with a history of transverse myelitis

**DOI:** 10.1093/braincomms/fcaf417

**Published:** 2025-10-27

**Authors:** Paweł Jakuszyk, Piotr Szukało, Bartosz Kossowski, Maciej Juryńczyk

**Affiliations:** Laboratory of Brain Imaging, Nencki Institute of Experimental Biology, Polish Academy of Sciences, Warsaw 02-093, Masovia, Poland; Laboratory of Brain Imaging, Nencki Institute of Experimental Biology, Polish Academy of Sciences, Warsaw 02-093, Masovia, Poland; Laboratory of Brain Imaging, Nencki Institute of Experimental Biology, Polish Academy of Sciences, Warsaw 02-093, Masovia, Poland; Laboratory of Brain Imaging, Nencki Institute of Experimental Biology, Polish Academy of Sciences, Warsaw 02-093, Masovia, Poland

**Keywords:** AQP4-NMOSD, magnetic resonance imaging, neurodegeneration, transverse myelitis

## Abstract

Neuromyelitis optica spectrum disorder associated with aquaporin-4-antibodies (AQP4-NMOSD) is an autoimmune disease of the CNS with a high risk of visual, motor and sensory disability secondary to optic neuritis (ON) and transverse myelitis (TM) attacks. The degree of recovery is difficult to predict and may be affected by the extent of ensuing neurodegeneration. While neurodegeneration in AQP4-NMOSD is reported in the visual system after ON, its occurrence in patients with TM remains largely unknown. The aim of this study was to use advanced MRI to cross-sectionally examine the sensory and motor pathways in 18 AQP4-NMOSD patients with a history of TM and 20 healthy controls. The results showed that AQP4-NMOSD patients had decreased cross-sectional area (mean 63.62 versus 70.75, *P* = 0.016) and reduced fractional anisotropy (mean 0.60 versus 0.65, *P* = 0.014) in the cervical spinal cord, and changes in the sensory, but not motor, cerebral pathway as evidenced by higher isotropic volume fraction in the ventral posterolateral (VPL) nuclei (mean 0.06 versus 0.05, *P* = 0.03), reduced neurite density in the right superior thalamic radiation and lower T1 relaxation rates in the primary somatosensory cortex (mean 0.72 versus 0.74, *P* = 0.04) when compared with healthy controls. Neurite density in the VPL nuclei significantly correlated with the Expanded Disability Status Scale (*r* = −0.469, *P* < 0.05). In conclusion, AQP4-NMOSD patients who had TM display features of anterograde and trans-synaptic neurodegeneration in the sensory pathway, which correlate with clinical outcomes. Further studies will clarify the temporal dynamics of such changes and their potential utility as clinical trial outcomes.

## Teaser

Jakuszyk et al. report that patients with neuromyelitis optica spectrum disorder associated with aquaporin-4 antibodies and a history of transverse myelitis show neurodegeneration in sensory pathways, including spinal cord white matter and thalamocortical regions. Advanced MRI findings correlate with disability, suggesting biomarkers for disease progression and treatment evaluation.

## Graphical Abstract

**Graphical Abstract:**
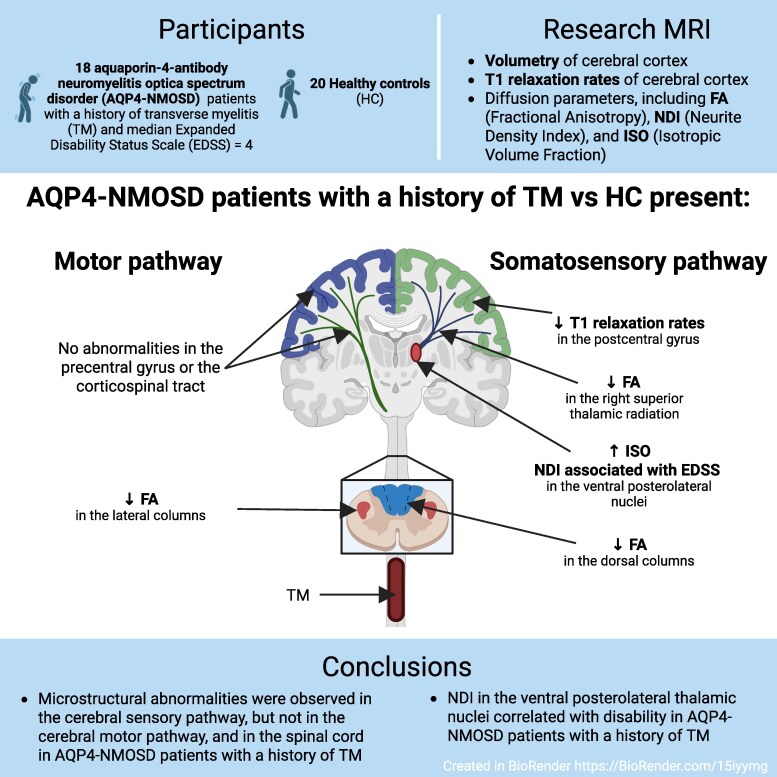

## Introduction

Neuromyelitis optica spectrum disorder (NMOSD) associated with aquaporin-4 (AQP4)-IgG is an autoimmune inflammatory disease of the CNS characterized predominantly by optic neuritis (ON), transverse myelitis (TM) and brain/brainstem attacks.^1^ AQP4-IgG plays a pathogenic role in NMOSD by targeting AQP4 located on astrocyte end-foot processes, which activates complement and leads to astrocyte damage, influx of inflammatory cells, secondary demyelination and neurodegeneration.^2^ AQP4-NMOSD attacks are typically highly disabling and the recovery is often partial or poor.^3^ The disability is attack-related and may accumulate with relapses. In a study including 106 AQP4-NMOSD patients from the UK and Japan followed for a median of 75 months, 18% of patients had permanent bilateral visual impairment, 34% were unable to walk further than 100 m unaided, 23% required a wheelchair for mobility and 9% had died.^4^

TM is the hallmark of NMOSD and the main cause of residual motor impairment and pain.^4^ It typically presents as longitudinally extensive transverse myelitis (LETM), which is defined radiologically by hyperintense lesions on T2-weighted imaging extending continuously over at least three segments of the spinal cord with cord swelling.^5,6^ Short-segment TM can also occur but is rare.^7^ The acutely inflamed spinal cord in AQP4-NMOSD typically undergoes focal atrophy extending over several segments.^8^ Advanced magnetic resonance imaging (MRI) demonstrates alterations in tissue integrity, evidenced by decreased white matter fractional anisotropy (FA) within the site of injury.^9,10^ While locally destructive, little is known about whether TM in AQP4-NMOSD is associated with any neurodegenerative process in the brain and if so whether such a process may be associated with clinical outcome. A study by Papadopoulou *et al*.^11^ has investigated the volume changes of ventral posterior thalamic nuclei, which receive afferent white matter fibres from the spinal cord; however, no significant volume loss in AQP4-NMOSD patients with a history of TM was observed.

**Figure 1:**
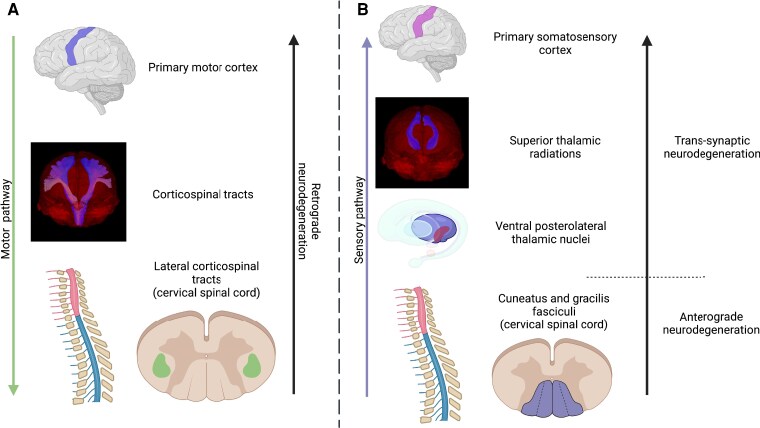
**Illustration of motor and sensory pathway structures assessed in this study using advanced MRI.** (**A**) The descending motor pathway: the primary motor cortex (precentral gyrus), cerebral corticospinal tract, lateral corticospinal tract in the cervical spinal cord. (**B**) The ascending sensory pathway: the posterior column (cuneatus and gracilis fasciculi) of the cervical spinal cord, the VPL thalamic nuclei, superior thalamic radiations and the primary somatosensory cortex (postcentral gyrus). Created in BioRender. J, P. (2025) https://BioRender.com/y99c691. VPL, ventral posterolateral.

In this prospective study, including patients with a history of TM due to AQP4-NMOSD, we applied advanced MRI to study microstructural changes in the motor and somatosensory pathways in the spinal cord and the brain, in particular: (i) lateral and posterior columns in the cervical spinal cord (using diffusion tensor imaging, DTI), (ii) thalamic VPL nuclei (volumetry and diffusion-based neurite orientation dispersion and density model, NODDI), (iii) the cerebral corticospinal tract and superior thalamic radiation (NODDI model) and (iv) primary motor (precentral gyrus) and somatosensory (postcentral gyrus) cortex (by assessing cortical thickness and T1 relaxation rates, Fig. 1). We also assessed whether structural MRI changes correlate with patients’ disability.

## Materials and methods

### Participants

This prospective cohort study received approval from the bioethics committee at the Institute of Psychiatry and Neurology in Warsaw, Poland (reference number 8/2021). Written informed consent was obtained from all participants. AQP4-NMOSD patients (*n* = 18) were recruited through the authors’ clinical neurology practice (M.J.) and via collaboration with neurologists at three Warsaw hospitals: Institute of Psychiatry and Neurology, Wolski Hospital and Medical University Hospital. The inclusion criteria required: (i) fulfilment of the revised 2015 diagnostic criteria for AQP4-NMOSD,^1^ (ii) a positive AQP4-IgG serostatus confirmed by a fixed cell-based assay and (iii) clinical remission for a minimum of two months prior to MRI scanning. The exclusion criteria included any contraindications to MRI scanning (e.g. claustrophobia, metal implants, pregnancy) and the absence of confirmed TM in AQP4-NMOSD patients. A cohort of healthy controls (HC) (*n* = 20), matched for age and sex with the AQP4-NMOSD group, was also recruited. HC were screened to exclude any neurological or other medical conditions that could confound the MRI findings, and the same MRI-related exclusion criteria were applied as for the patient group. Clinical information, including Expanded Disability Status Scale (EDSS) scores, and the timing, location, number and segmental extent of past TM attacks, was obtained from clinical notes as described previously.^12^

### Brain and cervical spinal cord MRI image acquisition

All study participants were scanned in the Laboratory of Brain Imaging, Nencki Institute of Experimental Biology, Polish Academy of Sciences in Warsaw. All MRI data were acquired using a 3 T Siemens Trio scanner (Siemens Erlangen, Germany). Due to the need to swap receiver coils, the research scan was divided into two sessions: brain and cervical spinal cord imaging, both conducted during a single visit with ∼15 min breaks between sessions. Brain MRI data were acquired with a 32-channel array head coil. The brain MRI protocol included: (i) T1-weighted magnetization-prepared rapid acquisition gradient echo (MPRAGE): repetition time/echo time/inversion time = 2530/3.32/1100 ms, acquisition time = 6 min; (ii) multi-shell diffusion-weighted sequence (DWI): repetition time/echo time/resolution = 3660/101 ms/2 × 2 × 2 mm^3^ isotropic with *b*-values of 0/500/1250/2500 s/mm^2^ and 13/18/36/53 measurements per shell, respectively, and an additional diffusion acquisition with seven measurements of *b*-value 0 s/mm^2^ with reversed phase encoding direction to correct for susceptibility-induced distortions, acquisition time = 8 min; (iii) T1-weighted MPRAGE echo with two separate readouts at different inversion times (MP2RAGE): repetition time/echo time/inversion time 1/inversion time 2 = 5000/2.96/700/2500 ms, and 1 mm^3^ isotropic spatial resolution, acquisition time = 8 min.

During the cervical spinal cord imaging session all participants were scanned using a 12-channel array head and neck coil. Spinal cord images were acquired according to the Spinal Cord Toolbox (SCT) standard operating procedure.^13^ The protocol included: (i) T2-weighted sequence: repetition time/echo time = 2800/83 ms, with 0.8 × 0.6 × 3.0 mm^3^ spatial resolution, acquisition time = 4.5 min; (ii) T1-weighted sequence: repetition time/echo time = 2000/3.21 ms, with 1 mm^3^ isotropic spatial resolution, acquisition time = 5 min; (iii) T2-weighted sequence: repetition time/echo time = 2000/118 ms, with 0.8 mm^3^ isotropic spatial resolution, acquisition time = 4.5 min; (iv) DWI: repetition time/echo time/ = 600/99 ms, 0.9 × 0.9 × 5 mm^3^ spatial resolution, with *b*-values of 0/800 s/mm^2^, 1/64 measurements per shell, respectively, and an additional diffusion acquisition with 4 measurements of *b*-value = 0 s/mm^2^ with reversed phase encoding direction to correct for susceptibility-induced distortions, acquisition time = 4 min; (v) spoiled T2* Multi-Echo Data Image Combination (MEDIC) sequence: repetition time/echo time = 600/13 ms, with 0.5 mm^3^ isotropic spatial resolution, acquisition time = 5 min. Due to participant withdrawal, the cervical spinal cord protocol was not performed for one HC participant.

### Cervical spinal cord image analysis

Cervical cord T2-weighted images were used to obtain a mean cross-sectional area (CSA). T2 images were automatically segmented to generate a binary spinal cord mask using the DeepSeg tool from SCT and manually adjusted if necessary. Subsequently, vertebral disc labelling was manually performed at the intervertebral level (posterior tip of each disc). Segmented spinal cord and vertebral labels were then used to transform the images to the PAM-50 spinal cord template.^14^ Quality of segmentation and registration process was ensured by visually inspecting each step using the quality control tool provided by SCT. CSA was computed at each vertebral level from C2 to C7, and the mean CSA used for statistical analysis was obtained by averaging the values across these six spinal cord segments (Supplementary Fig. 2).

Spinal cord DWI data were acquired with a field of view spanning C2 to C5 spinal cord segments. DWI data preprocessing was carried out according to standard SCT protocol and involved correction for susceptibility-induced distortions and motion. Subsequently, the DWI images were registered to the PAM-50 template using a multimodal registration algorithm. The DTI model was then fitted to the pre-processed DWI data resulting in generation of FA maps.

To assess FA within the normal-appearing white matter (NAWM) of the spinal cord, binary lesion masks were generated using the DeepSegLesion algorithm on axial MEDIC images and co-registered to DWI images. T2-weighted and T1-weighted images were also reviewed to confirm lesion locations within the cervical spine, and manual adjustments to the lesion masks were performed when necessary. To ensure that only unaffected tissue was analysed, the lesion masks were subtracted from the co-registered white matter atlas to generate individual NAWM masks for each participant. The mean FA was then calculated exclusively within these NAWM masks, explicitly excluding all lesioned areas. FA was also calculated for motor and sensory spinal cord tracts obtained from the PAM-50 white matter atlas. The motor tracts included the left and right corticospinal tracts, while the sensory tracts included the left and right cuneate and gracile fasciculi (dorsal columns).

### Assessment of motor and sensory cerebral white matter tracts

DWI images were pre-processed according to standard MRtrix3 pipeline,^15^ which included: denoising, Gibbs artefact removal, motion correction, eddy-currents correction, echo-planar imaging distortions correction and intensity bias correction. Neurite density index (NDI), orientation dispersion index (ODI) and isotropic volume fraction (ISO) maps were generated by fitting the nonlinear three-compartment NODDI model to pre-processed DWI data using the NODDI Matlab Toolbox.^16^

To perform tract segmentation, response functions for white matter, grey matter and CSF were estimated and a multi-tissue constrained spherical deconvolution (CSD) algorithm was used to obtain white matter fibres orientation distribution.^17^ CSD peaks indicating the dominant orientations of fibres within a voxel were fed into TractSeg to generate white matter tract segmentations. Two efferent (left and right corticospinal tract) and two afferent (left and right superior thalamic radiation) white matter tracts segmented using TractSeg and subsequently analysed with its tractometry module (Supplementary Fig. 3). Additionally, two thalamic tracts not involved in sensation were investigated for comparison (left and right thalamo-parietal tracts). Tracseg’s tractometry module, a technique that quantifies diffusion microstructural properties along the length of specific white matter tracts to enable localized analysis of tissue integrity, was used to evaluate NDI along the selected tracts. For each tract, a central line was computed, and the streamlines were divided into 100 equidistant segments. NDI values were sampled along these segments, and a permutation-based statistical test was applied to assess between-group differences at each segment.^18^

### Assessment of the VPL thalamic nuclei

Freesurfer’s subregion segmentation tool was used to obtain volumes of the thalamic nuclei. VPL nuclei volumes were calculated for each hemisphere and averaged. The assessed VPL nuclei included the medial portion, corresponding to the ventral posteromedial nuclei.^19^ Quality checks were performed by visually inspecting segmentation results for each participant. To assess microstructure within the VPL the nuclei masks obtained from Freesurfer’s segmentation were co-registered to DWI images and mean VPL values for NDI, ODI and ISO were calculated.

### Assessment of the motor and somatosensory cerebral cortex

Freesurfer segmentation software was used to calculate cortical thickness.^20^ T1-weighted MPRAGE images were pre-processed using the recon-all routine. Cortical thickness estimations were extracted for primary motor and primary somatosensory areas, for each hemisphere, using the Desikan-Killiany atlas-based parcellations.^21^ Values from both hemispheres were averaged.

T1 relaxation rates for the cerebral cortex were acquired with MP2RAGE. The T1 relaxation maps were registered to Freesurfer’s reconstruction using the previously denoised UNIFIED images. The Ciftify tool from the Human Connectome Project (HCP) was utilized to generate a grey matter ribbon. Subsequently, ribbon-constrained sampling and smoothing from the HCP Workbench were employed to project T1 relaxation rates onto the cortical surface.^22^ The averaged T1 relaxation rate estimates were extracted for primary motor and primary somatosensory cortical areas.

### Statistical analysis

Normality and equality of variance were tested for all variables. Differences in demographic characteristics were assessed with the independent samples *t*-test and *χ*^2^ test. Analyses of covariance (ANCOVA) with age and sex as covariates were conducted to assess FA in spinal cord afferent and efferent white matter tracts, CSA, NAWM, FA, VPL nuclei volumes, VPL nuclei microstructure, cortical thickness and T1 relaxation of the primary motor and primary somatosensory cortex. Cohen’s *d* (*d*) was calculated to assess the effect size of all aforementioned tests. Tractometry analysis along the selected white matter tracts was performed using a permutation-based method, which involved randomly shuffling group labels across participants to generate a null distribution of *t*-test statistics for each segment comparison. This approach allowed for robust inference while controlling for multiple comparisons along the tract. The analysis was adjusted for age and sex. Correlations between advanced MRI measures and clinical parameters were assessed using Pearson’s *r*.

## Results

### Sample demographics, clinical and basic imaging characteristics

AQP4-NMOSD patients had a median EDSS score of 4 (1–7.5) and a mean disease duration of 8.8 (0.5–29) years. They had a median of 2.5 (1–7) TM attacks and were scanned on average 45.5 (4–91) months after their most recent TM attack. The median maximum number of spinal cord segments involved per TM attack was 7 (1–17). Sixteen AQP4-NMOSD patients had a history of LETM, and 2 had short-segment TM only. In 12 patients, past documented TM lesions were located in both the cervical and thoracic cord, while six patients had a history of thoracic TM only. Six AQP4-NMOSD patients had at least 1 residual T2 hyperintense lesion in the cervical spinal cord on the research scan (Table 1).

**Table 1:** Sample demographics and clinical characteristics

	AQP4-NMOSD patients	HC
Sex, *n* (male/female)	18 (2/16)	20 (2/18)
Age, years, mean ± SD	50 ± 13	49 ± 8
EDSS score, median (range)	4 (1–7.5)	—
Number of TM attacks, median (range)	2.5 (1–7)	—
Disease duration, years, mean (range)	8.8 (0.5–29)	—
Time from last TM attack to study scan, months, mean (range)	45,5 (4–91)	—
Maximum number of segments involved in a single TM attack, median (range)	7 (1–17)	—
LETM type TM presentation, *n*	16	—
Short-segment TM presentation, *n*	2	—
Cervical cord lesion present, *n*	6	—

NMOSD, neuromyelitis optica spectrum disorders; HC, healthy controls; AQP4, Aquaporin-4; EDSS, Expanded Disability Status Scale; *n*, number of participants; SD, standard deviation; TM, transverse myelitis; LETM, longitudinally extensive transverse myelitis.

### Atrophy and white matter damage in the cervical spinal cord

**Figure 2:**
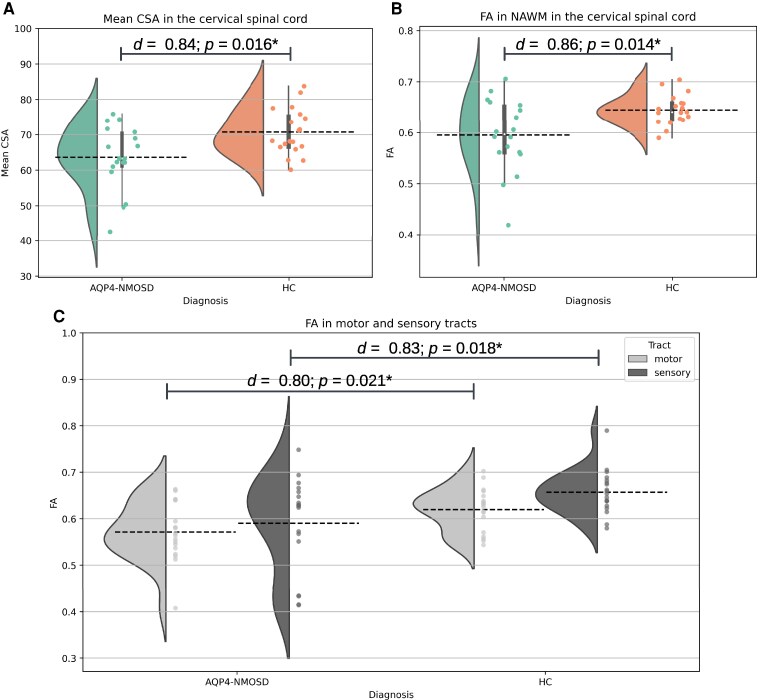
**Volumetric and microstructural differences between AQP4-NMOSD patients with a history of TM and HC in the cervical spinal cord.** ANCOVA with age and sex as covariates was used to compare HC (*N* = 20) with AQP4-NMOSD patients (*N* = 18) who showed: (**A**) lower mean CSA values in the cervical spinal cord (*P* = 0.016), (**B**) lower mean FA in NAWM in the cervical spinal cord (*P* = 0.014) and (**C**) lower mean FA in sensory (*P* = 0.018) and motor (*P* = 0.021) tracts. AQP4, aquaporin-4; NMOSD, neuromyelitis optica spectrum disorders; HC, healthy controls; TM, transverse myelitis; CSA, cross-sectional area; FA, fractional anisotropy; NAWM, normal-appearing white matter; *d*, Cohen’s *d*.

When compared with HC, AQP4-NMOSD patients had significantly reduced mean CSA (AQP4-NMOSD *M* = 63.62, *SD* = 9.00; HC *M* = 70.75, *SD* = 6.54; *d*  *=* 0.84, 95% CI: 0.13, 1.55, *P* = 0.016; Fig. 2A) and FA in the spinal cord NAWM (AQP4-NMOSD *M* = 0.60, *SD* = 0.07; HC *M* = 0.65, *SD* = 0.07; *d*  *=* 0.86, 95% CI: 0.15, 1.57, *P* = 0.014; Fig. 2B). To examine whether the damage was restricted to any particular tracts we have then assessed FA separately in sensory (dorsal columns) and motor (lateral corticospinal) tracts and found significant decrease in both (sensory tracts AQP4-NMOSD *M* = 0.59, SD = 0.10; HC *M* = 0.65, SD = 0.05, *d*  *=* 0.83, 95% CI: 0.12, 1.53, *P* = 0.018; motor tracts AQP4-NMOSD *M* = 0.57, SD = 0.06; HC *M* = 0.62, SD = 0.05; *d*  *=* 0.80, 95% CI: 0.10, 1.50, *P* = 0.021; Fig. 2C).

### No evidence of neurodegeneration in the cerebral motor pathway

We have not identified any significant difference in neurite density between AQP4-NMOSD patients and HC in any of the 100 sampled segments of neither left nor right corticospinal tract (Supplementary Fig. 1). Moreover, the primary motor cortex in the AQP4-NMOSD patients did not differ in thickness (*M* = 2.57, SD = 0.08 versus. *M* = 2.61, SD = 0.20) nor T1 relaxation rates (*M* = 0.75, SD = 0.02 versus. *M* = 0.75, SD = 0.02) when compared with HC.

### Altered microstructure in the cerebral sensory pathway

**Figure 3:**
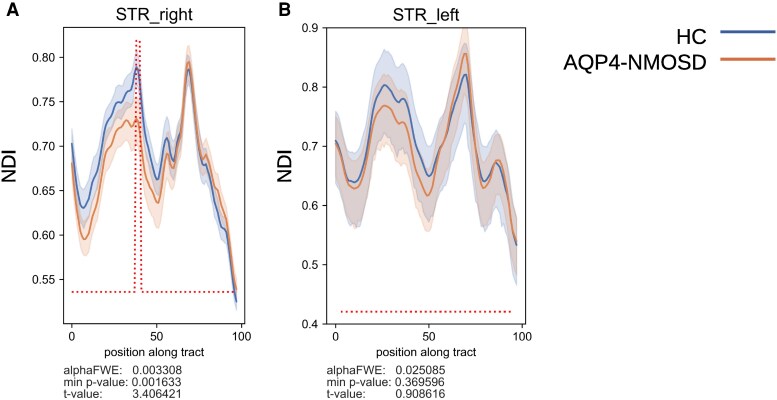
**Tractometry analysis of NDI in (A) right and (B) left superior thalamic radiations.** Permutation-based *t*-tests showed significant NDI differences between AQP4-NMOSD patients (*N* = 18) and HC (*N* = 20). The elevated dotted line in the right superior thalamic radiations indicates segments where the NDI is significantly lower in patients with AQP4-NMOSD compared to HC. The starting position (point 0) along the tract corresponds to the area of the tract closest to the cerebral cortex, while the ending position (point 100) represents the region closest to the thalamus. AQP4, aquaporin-4; NMOSD, neuromyelitis optica spectrum disorders; NDI, neurite density index; HC, healthy controls; STR, superior thalamic radiations; FWE, family-wise error.

We have next assessed the cerebral sensory pathway by investigating integrity in the thalamic VPL nuclei (volume, NDI, ODI, ISO), superior thalamic white matter tract (NDI) and primary somatosensory cortex (thickness, T1 relaxation rates) in AQP4-NMOSD patients and HC. In the thalamus, we have found increased ISO but preserved NDI, ODI and volume in the VPL nuclei of AQP4-NMOSD patients when compared to HC (Table 2). AQP4-NMOSD patients also had significantly reduced NDI in the right superior thalamic radiation (*P* = 0.002, Fig. 3). No significant NDI changes were found in non-sensory thalamo-parietal tracts (data not shown). Moreover, in the primary somatosensory cortex T1 relaxation rates, but not cortical thickness, were lower in AQP4-NMOSD patients when compared with HC (Table 2).

**Table 2:** MRI metrics of the assessed cerebral sensory grey matter structures

	AQP4-NMOSD patients	HC	Cohens’ d	95% CI		*P*-value
				Lower	Upper	
**VPL**						
NDI, mean ± SD	0.58 ± 0.02	0.58 ± 0.02	—	—	—	0.52
ISO, mean ± SD	0.06 ± 0.02	0.05 ± 0.01	0.73	0.05	1.42	**0**.03*
ODI, mean ± SD	0.32 ± 0.03	0.33 ± 0.02	—	—	—	0.36
Volume, mean ± SD	0.06 ± 0.01	0.05 ± 0.01	—	—	—	0.75
**Primary somatosensory cortex**						
Thickness, mean ± SD	2.04 ± 0.10	2.10 ± 0.14	—	—	—	0.15
T1 relaxation rate, mean ± SD	0.72 ± 0.02	0.74 ± 0.01	0.66	0.02	1.35	**0**.04*

NMOSD, neuromyelitis optica spectrum disorders; HC, healthy controls; AQP4, aquaporin-4; SD, standard deviation; VPL, ventral posterolateral thalamic nuclei; NDI, neurite density index; ISO, isotropic volume fraction; ODI, orientation dispersion index; CI, confidence interval. Bold, *P-value < 0.05.

### Association with clinical parameters

**Figure 4:**
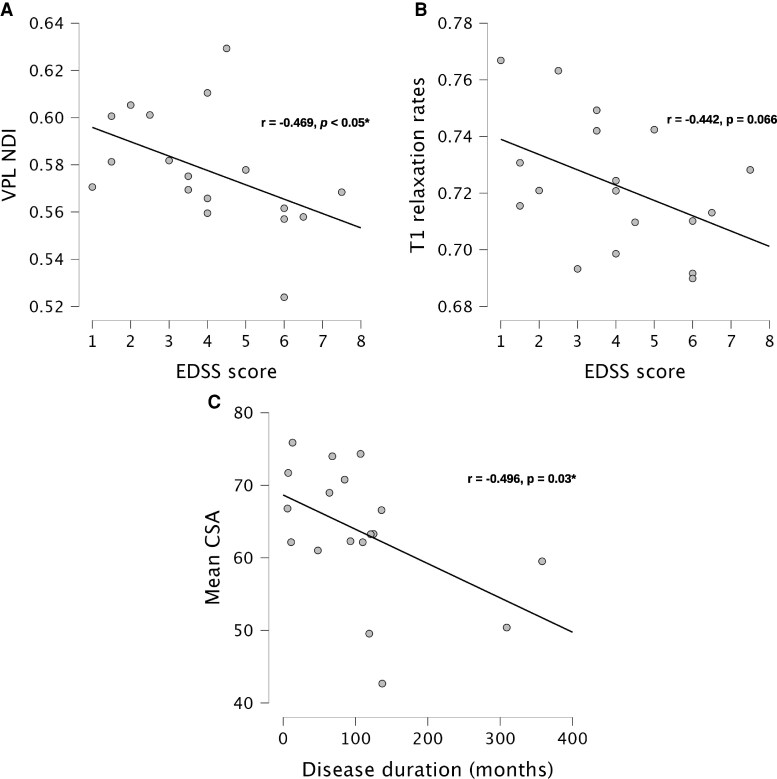
**Correlations between MRI sensory pathway metrics and clinical measures in AQP4-NMOSD patients.** Pearson’s correlation for AQP4-NMOSD patients (*N* = 18) showed: (**A**) Significant negative correlation between NDI in VPL thalamic nuclei and EDSS (*r* = –0.469, *P* < 0.05), (**B**) negative correlation between T1 relaxation rates in the primary somatosensory cortex and EDSS (*r* = −0.442, *P* = 0.066) and (**C**) significant negative correlation between mean CSA and disease duration (months) (*r* = − 0.496, *P* = 0.03). AQP4, aquaporin-4; NMOSD, neuromyelitis optica spectrum disorders; NDI, neurite density index; VPL, ventral posterolateral; EDSS, expanded disability status scale; CSA, cross-sectional area.

When examining correlations between MRI measures and disability in AQP4-NMOSD patients, higher EDSS was associated with lower NDI in the VPL (*r* = −0.469, *P* < 0.05, Fig. 4A), and there was also a non-significant trend toward lower T1 relaxation rates in the primary somatosensory cortex (*r* = −0.442, *P* = 0.066, Fig. 4B). For other clinical measures of disease burden, including disease duration, number of TM episodes, maximum number of spinal segments involved in a single attack and time since the last TM attack, the only significant finding was a negative correlation between disease duration and mean CSA (*r* = −0.49, *P* = 0.03, Fig. 4C).

## Discussion

In this prospective MRI study, we have used advanced cervical spinal cord and brain imaging techniques to assess spinal cord and cerebral motor and sensory pathways in AQP4-NMOSD patients with a history of TM. First of all, we have identified significant cervical spinal cord atrophy and spinal cord white matter damage in AQP4-NMOSD patients who had TM when compared with HC. The spinal cord damage was evident both in the lateral and posterior columns prompting us to assess the ascending and descending pathways in the brain. We have found evidence of structural damage in the sensory pathway at multiple sites in the spinal cord and in the brain. In particular, AQP4-NMOSD patients had higher ISO in thalamic VPL, lower NDI in the right superior thalamic radiation and lower T1 relaxation rates in the primary somatosensory cortex. Moreover, structural alterations in the cerebral sensory pathway were associated with disability outcome, which was not observed for spinal cord measures.

While the history of TM in our study was associated with both micro- and macrostructural changes in the cervical spinal cord of AQP4-NMOSD patients, damage to the cerebral sensory pathway was only identified on the microstructural level, likely corresponding to reduced fibre density and myelin content.^23,24^ Mild to moderate level of tissue disintegrity in the cerebral sensory pathway was also recently reported in patients with degenerative cervical myelopathy.^25^ More pronounced anterograde and trans-synaptic neurodegeneration in the sensory pathway involving thalamic atrophy and cortical atrophy have been evidenced in patients who had spinal cord injury.^26-29^

Despite marked motor disability in our AQP4-NMOSD cohort (median EDSS of 4) in our study we have not identified any alterations in the cerebral motor pathway. This is in line with previous research demonstrating the preservation of motor networks, as opposed to the somatosensory system in patients with traumatic spinal cord injury.^30,31^ Other studies in patients with traumatic spinal cord injury showed that retrograde degeneration occurs but decelerates over the two years after the acute phase.^32^ Since our patients were scanned on average more than 3 years after the last TM attack it is not excluded that due to adaptive changes no sustained alterations in the motor pathway could be identified. The severity of attacks and features specific to AQP4-NMOSD TM-induced injury might also play a role. Astrocytes are the selective target of immune attack in AQP4-NMOSD initiated by pathogenic antibodies activating complement with loss of immunoreactivity for astrocytic proteins being a pathological hallmark of the disease.^33^ Demyelination and axonal loss are secondary events and may vary in severity depending on the recruitment of specific immune cells, including macrophages and granulocytes and cytokines, including TNF-α and IL-6.^34,35^

Anterograde and trans-synaptic degeneration was previously reported in AQP4-NMOSD in patients who had ON and was demonstrated in the optic pathways at the level of retina, thalamus and visual cortex.^36,37^ Neurodegeneration in the visual system likely impacts retinal structure and visual function and has been hypothesized to serve as a marker of structural damage.^11^ Our study focusing on TM rather than ON-induced changes in AQP4-NMOSD complements these findings and offers further insights into attack-driven neurodegeneration in AQP4-NMOSD. Importantly, we have observed that in patients with a history of TM changes in the cerebral somatosensory system were significantly associated with disability on EDSS. A similar association was observed in patients with spinal cord injury, where a smaller decrease in magnetization transfer in the somatosensory cortex corresponded to a better functional independence.^29^ In the same study R2* relaxation rates in the secondary sensory cortices were associated with the intensity of neuropathic pain, which is also a frequent complication of AQP4-NMOSD likely influenced by the volume of VPL^38^ and the TM lesion level.^39^

A cross-sectional rather than longitudinal design is the main limitation of the study, as it proves only indirectly a causative relationship between TM and microstructural alterations in the sensory system. However, since these changes were demonstrated at multiple sites along the sensory pathway, were not observed elsewhere (corticospinal tracts, thalamic tracts not involved in sensation), and occurred in patients with severe spinal cord damage and no documented history of brain attacks, we argue that this effect is highly likely. This is further supported by significant association between the degree of somatosensory changes and clinical disability. A longitudinal research MRI study recruiting patients in the acute phase of TM is the next step forward but given the rarity of AQP4-NMOSD would require a multicentre collaboration with homogenized advanced MRI research protocols. As part of developing such protocols, particular attention should be given to the potential impact of asymptomatic brain lesions. Although none of the AQP4-NMOSD patients had a clinical history of brain attacks and the mean total brain lesion volume in our cohort was low, an unlikely contribution of asymptomatic brain lesions to the abnormalities in the cerebral sensory tract in patients with a history of TM cannot be entirely ruled out and could be considered a potential limitation of this study. Small sample size is also a limitation of this study and is due to the rarity of AQP4-NMOSD and the strict inclusion criterion of the previous history of TM. Furthermore, we acknowledge that our study did not include clinical measures specifically targeting somatosensory function and patient-reported outcomes, such as neuropathic pain assessments or EDSS functional system scores. Incorporating these measures in future longitudinal studies would provide a more comprehensive understanding of disease burden and better capture the patient experience in AQP4-NMOSD.

To our knowledge, this study is the first to comprehensively evaluate the ascending and descending pathways in AQP4-NMOSD patients who had TM. Our results suggest that these patients display mild to moderate anterograde and trans-synaptic degeneration in the sensory pathway accompanied by the preservation of neuronal integrity in the cerebral motor pathways. Further studies will need to elucidate the temporal dynamics of TM-induced processes in AQP4-NMOSD and address whether MRI changes in the somatosensory system could serve as an objective outcome measure in clinical trials with strategies aiming at improving the recovery of patients, including drugs targeting repair of the spinal cord, a major unmet need in AQP4-NMOSD.

## Supplementary Material

fcaf417_Supplementary_Data

## Data Availability

Data are available from the corresponding author upon request. Shell and Python scripts used in data preprocessing and analysis are located in the following GitHub repository https://github.com/nencki-lobi/NAWA/.

## References

[1] Wingerchuk DM, Banwell B, Bennett JL, et al International consensus diagnostic criteria for neuromyelitis optica spectrum disorders. Neurology. 2015;85(2):177–189. 26092914 10.1212/WNL.0000000000001729 PMC4515040

[2] Carnero CE, Correale J. Neuromyelitis optica spectrum disorders: From pathophysiology to therapeutic strategies. J Neuroinflammation. 2021;18(1):208. 34530847 10.1186/s12974-021-02249-1 PMC8444436

[3] Kleiter I, Gahlen A, Borisow N, et al Neuromyelitis optica: Evaluation of 871 attacks and 1,153 treatment courses. Ann Neurol. 2016;79(2):206–216. 26537743 10.1002/ana.24554

[4] Kitley J, Leite MI, Nakashima I, et al Prognostic factors and disease course in aquaporin-4 antibody-positive patients with neuromyelitis optica spectrum disorder from the United Kingdom and Japan. Brain. 2012;135(Pt 6):1834–1849. 22577216 10.1093/brain/aws109

[5] Ciccarelli O, Cohen JA, Reingold SC, Weinshenker BG. Spinal cord involvement in multiple sclerosis and neuromyelitis optica spectrum disorders. Lancet Neurol. 2019;18(2):185–197. 30663608 10.1016/S1474-4422(18)30460-5

[6] Juryńczyk M, Craner M, Palace J. Overlapping CNS inflammatory diseases: Differentiating features of NMO and MS. J Neurol Neurosurg Psychiatry. 2015;86(1):20–25. 25248365 10.1136/jnnp-2014-308984

[7] Huda S, Whittam D, Bhojak M, Chamberlain J, Noonan C, Jacob A. Neuromyelitis optica spectrum disorders. Clin Med. 2019;19(2):169–176. 10.7861/clinmedicine.19-2-169 PMC6454358 30872305

[8] Kim HJ, Paul F, Lana-Peixoto MA, et al MRI characteristics of neuromyelitis optica spectrum disorder. Neurology. 2015;84(11):1165–1173. 25695963 10.1212/WNL.0000000000001367 PMC4371410

[9] Mariano R, Messina S, Roca-Fernandez A, Leite MI, Kong Y, Palace JA. Quantitative spinal cord MRI in MOG-antibody disease, neuromyelitis optica and multiple sclerosis. Brain. 2021;144(1):198–212. 33206944 10.1093/brain/awaa347

[10] Lersy F, Noblet V, Willaume T, et al Identification and measurement of cervical spinal cord atrophy in neuromyelitis optica spectrum disorders (NMOSD) and correlation with clinical characteristics and cervical spinal cord MRI data. Rev Neurol (Paris). 2021;177(1–2):85–92. 32753321 10.1016/j.neurol.2020.05.007

[11] Papadopoulou A, Oertel FC, Gaetano L, et al Attack-related damage of thalamic nuclei in neuromyelitis optica spectrum disorders. J Neurol Neurosurg Psychiatry. 2019;90(10):1156–1164. 31127016 10.1136/jnnp-2018-320249

[12] Jakuszyk P, Podlecka-Piętowska A, Kossowski B, Nojszewska M, Zakrzewska-Pniewska B, Juryńczyk M. Patterns of cerebral damage in multiple sclerosis and aquaporin-4 antibody-positive neuromyelitis optica spectrum disorders—Major differences revealed by non-conventional imaging. Brain Commun. 2024;6(5):fcae295. 39258257 10.1093/braincomms/fcae295 PMC11384145

[13] Cohen-Adad J, Alonso-Ortiz E, Abramovic M, et al Generic acquisition protocol for quantitative MRI of the spinal cord. Nat Protoc. 2021;16(10):4611–4632. 34400839 10.1038/s41596-021-00588-0 PMC8811488

[14] De Leener B, Fonov VS, Collins DL, Callot V, Stikov N, Cohen-Adad J. PAM50: PAM50: Unbiased multimodal template of the brainstem and spinal cord aligned with the ICBM152 space. Neuroimage. 2018;165:170–179. 29061527 10.1016/j.neuroimage.2017.10.041

[15] Tournier JD, Smith R, Raffelt D, et al MRtrix3: A fast, flexible and open software framework for medical image processing and visualisation. Neuroimage. 2019;202:116137. 31473352 10.1016/j.neuroimage.2019.116137

[16] Zhang H, Schneider T, Wheeler-Kingshott CA, Alexander DC. NODDI: Practical in vivo neurite orientation dispersion and density imaging of the human brain. Neuroimage. 2012;61(4):1000–1016. 22484410 10.1016/j.neuroimage.2012.03.072

[17] Dhollander T, Connelly A. A novel iterative approach to reap the benefits of multi-tissue CSD from just single-shell (+b=0) diffusion MRI data. In: *Conference paper: 24th International Society of Magnetic Resonance in Medicine in Singapore*, Volume: 24, 3010. 2016.

[18] Wasserthal J, Maier-Hein KH, Neher PF, et al Multiparametric mapping of white matter microstructure in catatonia. Neuropsychopharmacology. 2020;45(10):1750–1757. 32369829 10.1038/s41386-020-0691-2 PMC7419514

[19] Iglesias JE, Insausti R, Lerma-Usabiaga G, et al A probabilistic atlas of the human thalamic nuclei combining ex vivo MRI and histology. NeuroImage. 2018;183:314–326. 30121337 10.1016/j.neuroimage.2018.08.012 PMC6215335

[20] Fischl B . FreeSurfer. NeuroImage. 2012;62(2):774–781. 22248573 10.1016/j.neuroimage.2012.01.021 PMC3685476

[21] Desikan RS, Ségonne F, Fischl B, et al An automated labeling system for subdividing the human cerebral cortex on MRI scans into gyral based regions of interest. NeuroImage. 2006;31(3):968–980. 16530430 10.1016/j.neuroimage.2006.01.021

[22] Connectome—Workbench Commands . Accessed 12 January 2025. https://www.humanconnectome.org/software/workbench-command/-volume-label-to-surface-mapping

[23] Rahmanzadeh R, Weigel M, Lu PJ, et al A comparative assessment of myelin-sensitive measures in multiple sclerosis patients and healthy subjects. Neuroimage Clin. 2022;36:103177. 36067611 10.1016/j.nicl.2022.103177 PMC9468574

[24] Kamiya K, Hori M, Aoki S. NODDI in clinical research. J Neurosci Methods. 2020;346:108908. 32814118 10.1016/j.jneumeth.2020.108908

[25] Freund P, Boller V, Emmenegger TM, et al Quantifying neurodegeneration of the cervical cord and brain in degenerative cervical myelopathy: A multicentre study using quantitativemagnetic resonance imaging. Eur J Neurol. 2024;31(7):e16297. 38713645 10.1111/ene.16297 PMC11235710

[26] Freund P, Weiskopf N, Ashburner J, et al MRI investigation of the sensorimotor cortex and the corticospinal tract after acute spinal cord injury: A prospective longitudinal study. Lancet Neurol. 2013;12(9):873–881. 23827394 10.1016/S1474-4422(13)70146-7 PMC3744750

[27] Freund P, Weiskopf N, Ward NS, et al Disability, atrophy and cortical reorganization following spinal cord injury. Brain. 2011;134(Pt 6):1610–1622. 21586596 10.1093/brain/awr093 PMC3102242

[28] Grabher P, Callaghan MF, Ashburner J, et al Tracking sensory system atrophy and outcome prediction in spinal cord injury. Ann Neurol. 2015;78(5):751–761. 26290444 10.1002/ana.24508 PMC4737098

[29] Ziegler G, Grabher P, Thompson A, et al Progressive neurodegeneration following spinal cord injury. Neurology. 2018;90(14):e1257–e1266. 29514946 10.1212/WNL.0000000000005258 PMC5890610

[30] Jurkiewicz MT, Crawley AP, Verrier MC, Fehlings MG, Mikulis DJ. Somatosensory cortical atrophy after spinal cord injury: A voxel-based morphometry study. Neurology. 2006;66(5):762–764. 16534122 10.1212/01.wnl.0000201276.28141.40

[31] Crawley AP, Jurkiewicz MT, Yim A, et al Absence of localized grey matter volume changes in the motor cortex following spinal cord injury. Brain Res. 2004;1028(1):19–25. 15518637 10.1016/j.brainres.2004.08.060

[32] Schading S, David G, Max ET, et al Dynamics of progressive degeneration of major spinal pathways following spinal cord injury: A longitudinal study. Neuroimage Clin. 2023;37:103339. 36758456 10.1016/j.nicl.2023.103339 PMC9939725

[33] Lucchinetti CF, Guo Y, Popescu BF, Fujihara K, Itoyama Y, Misu T. The pathology of an autoimmune astrocytopathy: Lessons learned from neuromyelitis optica. Brain Pathol. 2014;24(1):83–97. 24345222 10.1111/bpa.12099 PMC3905574

[34] Marignier R, Nicolle A, Watrin C, et al Oligodendrocytes are damaged by neuromyelitis optica immunoglobulin G via astrocyte injury. Brain. 2010;133(9):2578–2591. 20688809 10.1093/brain/awq177

[35] Zhang H, Bennett JL, Verkman AS. Ex vivo spinal cord slice model of neuromyelitis optica reveals novel immunopathogenic mechanisms. Ann Neurol. 2011;70(6):943–954. 22069219 10.1002/ana.22551 PMC3319401

[36] Manogaran P, Hanson JVM, Olbert ED, et al Optical coherence tomography and magnetic resonance imaging in multiple sclerosis and neuromyelitis Optica Spectrum disorder. Int J Mol Sci. 2016;17(11):1894. 27854301 10.3390/ijms17111894 PMC5133893

[37] Tian DC, Su L, Fan M, et al Bidirectional degeneration in the visual pathway in neuromyelitis optica spectrum disorder (NMOSD). Mult Scler J. 2018;24(12):1585–1593. 10.1177/1352458517727604 28823217

[38] Asseyer S, Kuchling J, Gaetano L, et al Ventral posterior nucleus volume is associated with neuropathic pain intensity in neuromyelitis optica spectrum disorders. Mult Scler Relat Disord. 2020;46:102579. 33296976 10.1016/j.msard.2020.102579

[39] Tackley G, Vecchio D, Hamid S, et al Chronic neuropathic pain severity is determined by lesion level in aquaporin 4-antibody-positive myelitis. J Neurol Neurosurg Psychiatry. 2017;88(2):165–169. 27884934 10.1136/jnnp-2016-314991

