# Supplementary material for: Anterograde and trans-synaptic neurodegeneration in aquaporin-4-antibody neuromyelitis optica spectrum disorder patients with a history of transverse myelitis
Source: Brain Commun. 2025 Oct 27;7(6):fcaf417. doi: 10.1093/braincomms/fcaf417 (PMC12596129; doi:10.1093/braincomms/fcaf417)
Supplement: fcaf417_Supplementary_Data [file fcaf417_supplementary_data.docx]

**
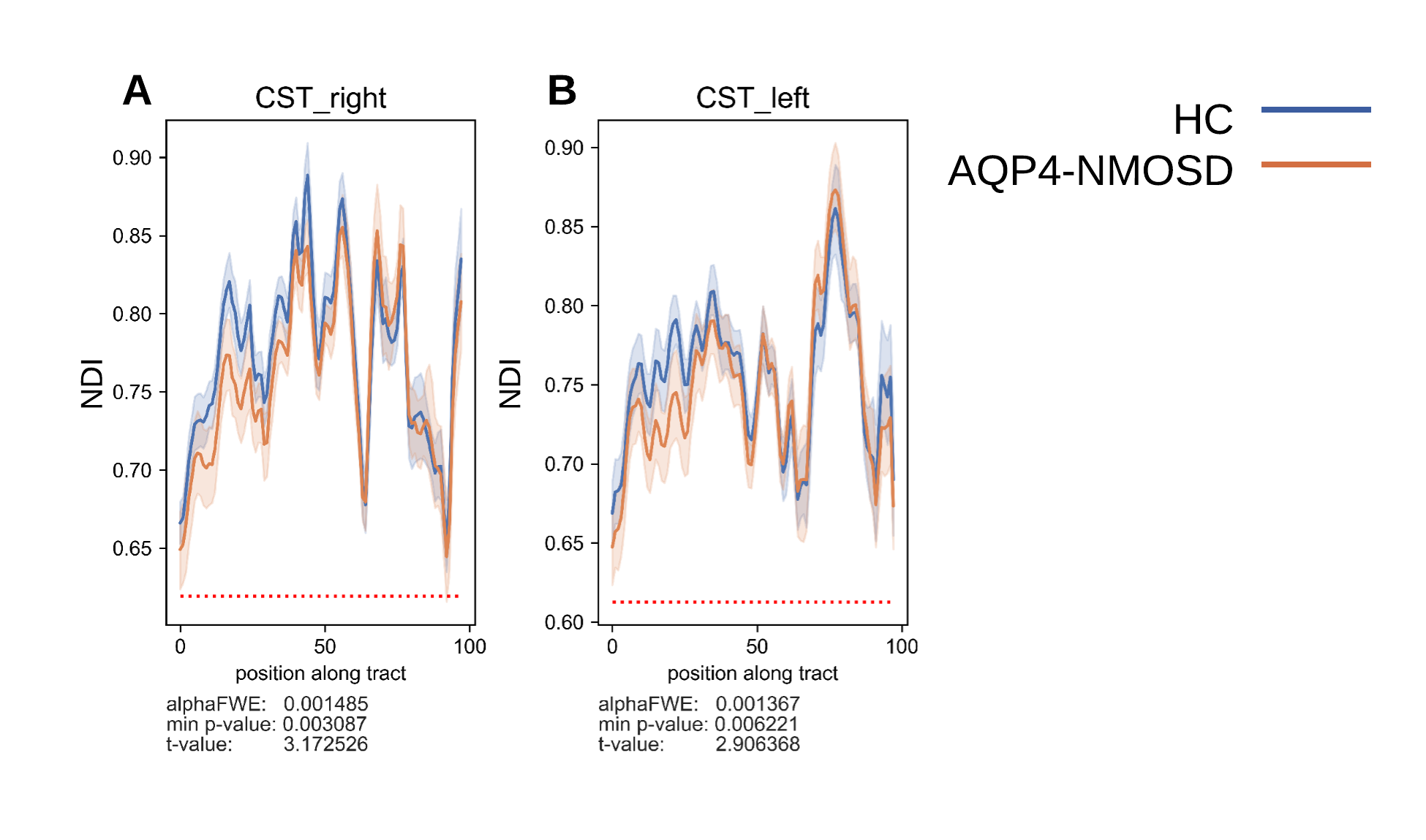
**

**Supplementary Figure 1: Tactometry analysis of NDI in (A) left and (B) right CST.** Permutation-based t-tests showed no significant NDI differences between AQP4-NMOSD patients (N = 18) and HC (N = 20) in any of the assessed tract segments. The starting position (point 0) along the tract corresponds to the area of the tract closest to the cerebral cortex, while the ending position (point 100) represents the region within the medulla. AQP4 = aquaporin-4; NMOSD = neuromyelitis optica spectrum disorders; HC = healthy controls; CST = corticospinal tracts, NDI = neurite density index, FWE = family-wise error.


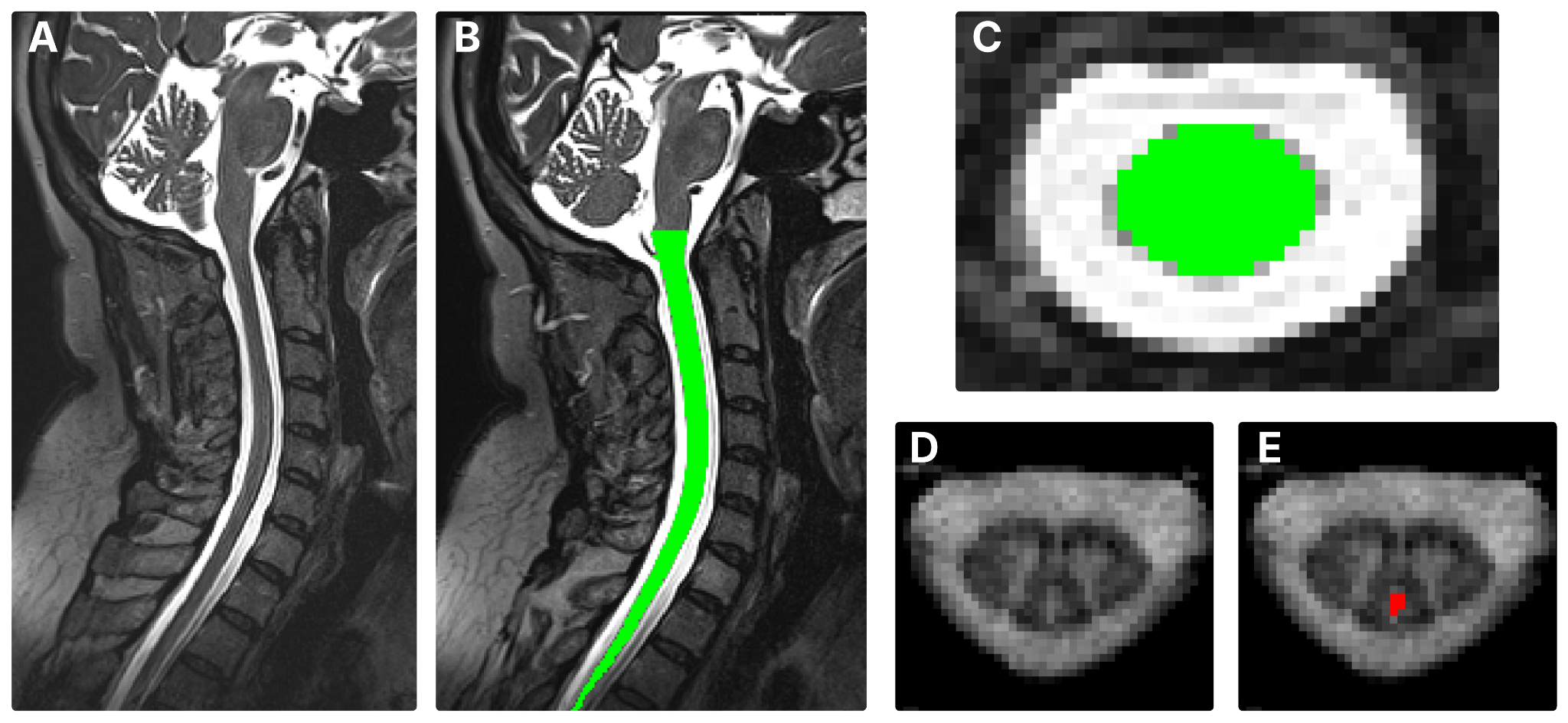


**Supplementary Figure 2: Examples of cervical spinal cord images and post-processing maps from a 54-year-old female patient with AQP4-NMOSD. (**A) Cervical spinal cord T2-weighted image with (B) sagittal and (C) axial images showing the segmented cord area used to calculate CSA. (D) Axial MEDIC image showing residual inflammatory lesion with lesion segmentation visible on (E). AQP4 = aquaporin-4; NMOSD = neuromyelitis optica spectrum disorders, CSA = cross-sectional area; MEDIC = multi-echo data image combination.


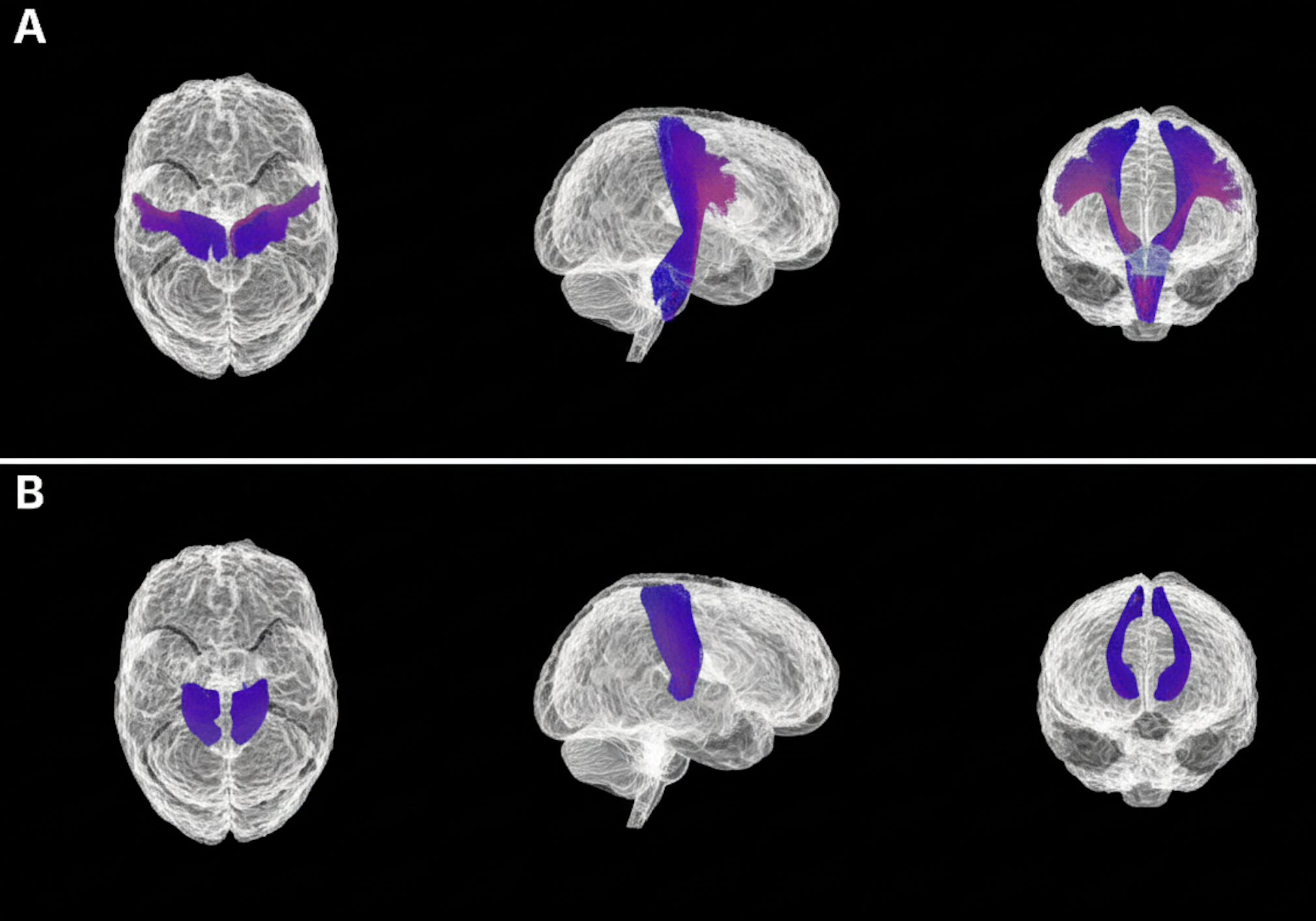


**Supplementary Figure 3: Examples of cerebral white matter tract segmentations depicted on a glass brain.** Axial, sagittal, and coronal views of **(**A) cerebrospinal tract, and (B) superior thalamic radiation.
